# Signal Space Separation Method for a Biomagnetic Sensor Array Arranged on a Flat Plane for Magnetocardiographic Applications: A Computer Simulation Study

**DOI:** 10.1155/2018/7689589

**Published:** 2018-04-26

**Authors:** Kensuke Sekihara

**Affiliations:** ^1^Signal Analysis Inc., Hachioji, Tokyo, Japan; ^2^Department of Advanced Technology in Medicine, Tokyo Medical and Dental University, 1-5-45 Yushima, Bunkyo-ku, Tokyo 113-8519, Japan

## Abstract

Although the signal space separation (SSS) method can successfully suppress interference/artifacts overlapped onto magnetoencephalography (MEG) signals, the method is considered inapplicable to data from nonhelmet-type sensor arrays, such as the flat sensor arrays typically used in magnetocardiographic (MCG) applications. This paper shows that the SSS method is still effective for data measured from a (nonhelmet-type) array of sensors arranged on a flat plane. By using computer simulations, it is shown that the optimum location of the origin can be determined by assessing the dependence of signal and noise gains of the SSS extractor on the origin location. The optimum values of the parameters *L_C_* and *L_D_*, which, respectively, indicate the truncation values of the multipole-order ℓ of the internal and external subspaces, are also determined by evaluating dependences of the signal, noise, and interference gains (i.e., the shield factor) on these parameters. The shield factor exceeds 10^4^ for interferences originating from fairly distant sources. However, the shield factor drops to approximately 100 when calibration errors of 0.1% exist and to 30 when calibration errors of 1% exist. The shielding capability can be significantly improved using vector sensors, which measure the *x*, *y*, and *z* components of the magnetic field. With 1% calibration errors, a vector sensor array still maintains a shield factor of approximately 500. It is found that the SSS application to data from flat sensor arrays causes a distortion in the signal magnetic field, but it is shown that the distortion can be corrected by using an SSS-modified sensor lead field in the voxel space analysis.

## 1. Introduction

Development of a sensor system that can measure biomagnetic signals in room-temperature environments has gained great interest. One promising candidate among room-temperature sensors for biomagnetic systems is magnetoresistive (MR) sensors [1–4], which can lead to the development of novel low-initial-cost and maintenance-free biomagnetic systems. A potential near-future application of such systems is a low-cost magnetocardiography (MCG) system using MR sensors [5]. Such low-cost and maintenance-free MCG systems could replace the 12-lead electrocardiogram (ECG) now routinely used in daily clinical examinations.

However, to develop such low-cost systems, one major problem is the removal of ambient noise magnetic fields that exist in urban hospital environments. Biomagnetic signals are many orders of magnitude weaker than these ambient interference magnetic fields, called environmental noise. To reduce the influence of such environmental noise, biomagnetic measurements have traditionally relied on two kinds of hardware-based solutions: one is magnetically shielded rooms (MSRs) and the other is gradiometer sensors [6].

According to a purely technical point of view, use of an MSR would be advantageous even for MR sensor systems. However, the use of a high- or a medium-quality MSR may invalidate our goal, which is developing low-initial-cost biomagnetic systems, because MSRs are, in general, very costly and the use of even a medium-quality MSR causes a considerable increase of the total initial cost of the system. Gradiometers can significantly reduce the environmental noise, and the reduction ratio of a typical first-order gradiometer is believed to reach almost two orders of magnitude [6], although the noise reduction capability strongly depends on the precision in sensor manufacturing. However, for MR sensor systems, it is currently unknown whether gradiometers can be incorporated into the MR sensor hardware design. No gradiometer-type MR sensors have been developed so far.

Therefore, to attain the goal of developing low-cost biomagnetic systems, it may not be possible to rely on traditional hardware-based methods. This paper addresses a third option, developing software shielding methods, namely, efficient signal processing methods for environmental noise cancellation. A number of signal processing methods have been developed for this purpose, and arguments for and against those methods can be found in [7]. This paper focuses on a method called signal space separation (SSS), which was originally proposed for environmental noise cancellation for magnetoencephalography (MEG) SQUID sensor arrays [8–10].

The SSS method has an excellent characteristic that it imposes almost no prerequisites on the data or sources (i.e., it does not use any restrictive data or source models). One mild prerequisite of the SSS method, which can naturally be fulfilled, is that the region in which sensors are installed should be source-free (i.e., no current sources in that region). Under this assumption, the method decomposes the measured data into two components originated from the so-called “internal” and “external” regions. The internal region refers to a region that is closer to the origin than the sensors are, and the external region refers to the one that is farther from the origin than the sensors are.

The method can efficiently suppress interferences overlapped onto biomagnetic signals if clever choices of the origin location can make the internal and external regions match the signal and interference regions, respectively. It is not difficult to find such origin locations for helmet-type sensor arrays used in MEG. However, the SSS method has not been considered applicable to data from nonhelmet-type sensor arrays, such as sensor arrays arranged on a flat plane, which are usually used for MCG systems, because it does not seem possible to find an appropriate origin location for those nonhelmet-type arrays.

This paper presents a computer simulation-based investigation that explores the possibility of applying the SSS method to data measured from an array of sensors arranged on a flat plane. The goal of the investigation is to show that the SSS method is still effective for data from such flat sensor arrays. A series of computer simulations are performed assuming flat sensor arrays whose sensor arrangement is typical in MCG applications [11–15]. Using the results of these computer simulations, this paper seeks optimal values for numerical parameters used in the SSS method, showing that their choices are crucial for the effective use of the SSS method when applied to flat sensor arrays. It is found that the application of the SSS method to flat sensor data causes a distortion of signals but that this distortion can be corrected by using the SSS-modified lead field in the voxel space analysis.

This paper is organized as follows. In Section 2, the SSS method is described in detail, including the analysis of the problems caused when the SSS method is applied to flat sensor data.  Section 3 presents computer simulation-based investigation, which shows the effectiveness of the SSS method for data from flat sensor arrays. This section includes empirical determinations of SSS parameters crucial to attain optimal performance of the SSS method.  Section 4 summarizes the findings of the investigation.

## 2. Signal Space Separation Method

### 2.1. Data Model

Biomagnetic measurement is conducted using a sensor array, which simultaneously measures the signal with multiple sensors. Let us define the measurement of the *m*th sensor as *y_m_*. The measurement from the whole sensor array is expressed as a column vector **y** : **y** = [**y**_1_, **y**_2_,…,**y**_*M*_]^*T*^. Here, *M* is the number of sensors, and the superscript *T* indicates the matrix transpose. Throughout this paper, plain italics indicates scalars, lowercase boldface indicates vectors, and uppercase boldface indicates matrices.

The location in the three-dimensional space is represented by **r** : **r** = (*x*, *y*, *z*). The source magnitude at **r** is denoted by a scalar *s*(**r**). The source vector is denoted by *s*(**r**), and the source orientation is denoted by **η** = [**η**_*x*_, **η**_*y*_, **η**_*z*_]^*T*^. We thus have the relationship: *s*(**r**) = *s*(**r**)**η**. Let us assume that a unit-magnitude source exists at **r**. When this unit-magnitude source is directed in the *x*, *y*, and *z* directions, the outputs of the *m*th sensor are, respectively, denoted by *l*_*m*_^*x*^(**r**), *l*_*m*_^*y*^(**r**) and *l*_*m*_^*z*^(**r**). Let us define an *M* × 3 matrix **L**(**r**) whose *m*th row is equal to [*l*_*m*_^*x*^(**r**), *l*_*m*_^*y*^(**r**), *l*_*m*_^*z*^(**r**)]. This matrix **L**(**r**), referred to as the lead field matrix, represents the sensitivity of the sensor array at **r**. When the unit-magnitude source at **r** is oriented in the **η** direction, the outputs of the sensor array are expressed as **l**(**r**) = **L**(**r**)**η**. This column vector **l**(**r**), referred to as the lead field vector, represents the sensitivity of the sensor array in the direction of **η** at the location **r**.

The outputs of the sensor array **y** are expressed as the sum of a magnetic signal **b** and additive sensor noise, represented by a random vector **ε**

(1) $$\begin{array}{c} y=b+\varepsilon . \end{array}$$

Here, the magnetic signal **b** is expressed as

(2) $$\begin{array}{c} b=b_{S}+b_{I}. \end{array}$$

Here, **b**_*S*_, called the signal vector, represents the biomagnetic signal that is the target of the measurements, and **b**_*I*_, called the interference vector, represents the interference overlapped onto the signal **b**_*S*_. In this paper, the interference **b**_*I*_ represents so-called environmental noise, and sources of environmental noise are assumed to be located much farther from the sensors than the sources of interest are. Sources of environmental noise are, in general, located from several meters (in cases of the noise sources such as electronic appliances in a laboratory) to several kilometers (in cases of urban environmental noise sources such as the subway noise) distant from the magnetically shielded room.

The sources that generate **b**_*S*_ are confined to a region called the source space (e.g., the source space is the cardiac region for MCG and the brain region for MEG measurements). Let us assume that a total of *Q* discrete sources exist in the source space. Their locations are denoted by **r**_1_,…, **r**_*Q*_, their orientations by **η**_1_,…, **η**_*Q*_, and their magnitudes by *s*_1_,…, *s*_*Q*_. Then, the source distribution is expressed as

(3) $$\begin{array}{c} s\left(r,t\right)=\overset{Q}{\underset{q=1}{\sum }}s_{q}\eta_{q}\delta \left(r-r_{q}\right), \end{array}$$

where *δ*(**r**) indicates the Dirac delta function. The signal vector **b**_*S*_ is expressed as

(4) $$\begin{array}{c} b_{S}=\int_{\Omega }L\left(r\right)\overset{Q}{\underset{q=1}{\sum }}s_{q}\eta_{q}\delta \left(r-r_{q}\right)dr=\overset{Q}{\underset{q=1}{\sum }}s_{q}l_{q}, \end{array}$$

where **l**_*q*_ represents the lead field vector of the *q*th source obtained such that **l**_*q*_ = **L**(**r**_*q*_)**η**_*q*_.

### 2.2. Derivation of SSS Basis Vectors

One fundamental assumption of the SSS method is that the sensors are installed in a source-free region, which is referred to as the sensor region. Then, the magnetic field at **r**, **B**(**r**), is expressed using the spherical polar coordinate *r* = (*r*, *θ*, *ϕ*) by

(5) $$\begin{array}{c} B\left(r\right)=-\mu_{0}\overset{\infty }{\underset{\ell =1}{\sum }}\overset{\ell }{\underset{m=-\ell }{\sum }}\alpha_{\ell ,m}\frac{\nu_{\ell ,m}\left(\theta ,\phi \right)}{r^{\ell +2}}-\mu_{0}\overset{\infty }{\underset{\ell =1}{\sum }}\overset{\ell }{\underset{m=-\ell }{\sum }}\beta_{\ell ,m}r^{\ell -1}\omega_{\ell ,m}\left(\theta ,\phi \right), \end{array}$$

where *μ*_0_ indicates the magnetic permeability of free space. In (5), **ν**_ℓ,*m*_(*θ*, *ϕ*) and **ω**_ℓ,*m*_(*θ*, *ϕ*) are the modified vector spherical harmonics [8, 16]. The index parameter ℓ is called the multipole-order or multipole parameter. In the right-hand side of (5), the first term represents the magnetic field generated from sources located closer to the origin than the sensors are.

The second term represents the magnetic field from sources located farther from the origin than the sensors are. The region closer to the origin than the sensors is referred to as the internal region, and the region farther from the origin than the sensors is referred to as the external region. Let us define the polar radial coordinate of the sensor nearest to the origin as *r*_*D*_^min^ and the radial coordinates of the sensor farthest from the origin as *r*_*D*_^max^. The internal region is formally defined as the region with *r* < *r*_*D*_^min^ and the external region as the region with *r* > *r*_*D*_^max^. The region with *r*_*D*_^min^ < *r* < *r*_*D*_^max^ is called the intermediate region.

Let us derive the SSS basis vectors. To do so, the magnetic signal detected by the *j*th sensor is denoted by *b*_*j*_ and the location and the normal vector of the *j*th sensor by *r*_*j*_ and *ζ*_*j*_. Then, we have

(6) $$\begin{array}{c} b_{j}=B\left(r_{j}\right)\cdot \zeta_{j}=b_{\mathrm{int}}^{j}+b_{\text{ext}}^{j}, \end{array}$$

where the notation “·” indicates taking the inner product between two vectors (note that when the area of the pickup coils is taken into consideration, the sensor signal *b*_*j*_ is obtained as a surface integral of **B**(**r**_*j*_) · **ζ**_*j*_ over the area of the *j*th pick-up coil). Here, *b*_int_^*j*^ and *b*_ext_^*j*^, respectively, represent magnetic components originating from the internal and external regions. These components are expressed as

(7) $$\begin{array}{c} b_{\mathrm{int}}^{j}=-\overset{\infty }{\underset{\ell =1}{\sum }}\overset{\ell }{\underset{m=-\ell }{\sum }}\alpha_{\ell ,m}\frac{\left[\nu_{\ell ,m}\left(\theta_{j},\phi_{j}\right)\cdot \zeta_{j}\right]}{r_{j}^{\ell +2}},\\ b_{\text{ext}}^{j}=-\overset{\infty }{\underset{\ell =1}{\sum }}\overset{\ell }{\underset{m=-\ell }{\sum }}\beta_{\ell ,m}r_{j}^{\ell -1}\left[\omega_{\ell ,m}\left(\theta_{j},\phi_{j}\right)\cdot \zeta_{j}\right], \end{array}$$

where we set *μ*_0_ = 1 for simplicity. Let us define the internal and external components of the vector **b** as **b**_int_ = [*b*_int_^1^,…,*b*_int_^*M*^]^*T*^ and **b**_ext_ = [*b*_ext_^1^,…,*b*_ext_^*M*^]^*T*^, which are expressed such that

(8) $$\begin{array}{c} b_{\mathrm{int}}=\overset{\infty }{\underset{\ell =1}{\sum }}\overset{\ell }{\underset{m=-\ell }{\sum }}\alpha_{\ell ,m}c_{\ell ,m},\\ b_{\text{ext}}=\overset{\infty }{\underset{\ell =1}{\sum }}\overset{\ell }{\underset{m=-\ell }{\sum }}\beta_{\ell ,m}d_{\ell ,m}, \end{array}$$

where column vectors **c**_ℓ,*m*_ and **d**_ℓ,*m*_ are given by

(9) $$\begin{array}{c} c_{\ell ,m}=\left[\begin{array}{c} \frac{1}{r_{1}^{\ell +2}}\left[\nu_{\ell ,m}\left(\theta_{1},\phi_{1}\right)\cdot \zeta_{1}\right]\\ \vdots \\ \frac{1}{r_{M}^{\ell +2}}\left[\nu_{\ell ,m}\left(\theta_{M},\phi_{M}\right)\cdot \zeta_{M}\right] \end{array}\right],d_{\ell ,m}=\left[\begin{array}{c} r_{1}^{\ell -1}\left[\omega_{\ell ,m}\left(\theta_{1},\phi_{1}\right)\cdot \zeta_{1}\right]\\ \vdots \\ r_{M}^{\ell -1}\left[\omega_{\ell ,m}\left(\theta_{M},\phi_{M}\right)\cdot \zeta_{M}\right] \end{array}\right]. \end{array}$$

Truncating the summation with respect to the multiple order ℓ to *L_C_* for **b**_int_ and *L_D_* for **b**_ext_, we finally obtain

(10) $$\begin{array}{c} b=b_{\mathrm{int}}+b_{\text{ext}}=\overset{L_{C}}{\underset{\ell =1}{\sum }}\overset{\ell }{\underset{m=-\ell }{\sum }}\alpha_{\ell ,m}c_{\ell ,m}+\overset{L_{D}}{\underset{\ell =1}{\sum }}\overset{\ell }{\underset{m=-\ell }{\sum }}\beta_{\ell ,m}d_{\ell ,m}. \end{array}$$

Thus, defining

(11) $$\begin{array}{c} C=\left[c_{1,-1},c_{1,0},c_{1,1},\ldots ,{{c_{L_{C}}}_{,}}_{L_{C}}\right],\\ D=\left[d_{1,-1},d_{1,0},d_{1,1},\ldots ,{{d_{L_{D}}}_{,}}_{L_{D}}\right],\\ \alpha ={\left[\alpha_{1,-1},\alpha_{1,0},\alpha_{1,1},\ldots ,{{\alpha_{L_{C}}}_{,}}_{L_{C}}\right]}^{T},\\ \beta ={\left[\beta_{1,-1},\beta_{1,0},\beta_{1,1},\ldots ,{{\beta_{L_{D}}}_{,}}_{L_{D}}\right]}^{T}, \end{array}$$

we obtain

(12) $$\begin{array}{c} b=b_{\mathrm{int}}+b_{\text{ext}}=C\alpha +D\beta =\left[C,D\right]\left[\begin{array}{l} \alpha \\ \beta \end{array}\right]=Sx, \end{array}$$

where **S** = [**C**, **D**] and **x** = [**α**^*T*^, **β**^*T*^]^*T*^. Here, **C** is an *M* × *N*_*C*_ matrix, and **D** is an *M* × *N*_*D*_ matrix, where

(13) $$\begin{array}{c} N_{C}=L_{C}^{2}+2L_{C},\\ N_{D}=L_{D}^{2}+2L_{D}. \end{array}$$

Note that the truncation values *L*_*C*_ and *L*_*D*_ correspond to the highest spatial frequencies possibly contained in **b**_int_ and **b**_ext_ [9, 17], respectively. Therefore, setting these parameters at too low values may result in an insufficient representation of the signal vectors **b**_int_ and **b**_ext_. The effects of *L*_*C*_ and *L*_*D*_ for data from the 306-channel Elekta Neuromag have been investigated and values of *L*_*C*_ = 8 and *L*_*D*_ = 3 were found to be sufficient for such data sets in [8, 9].

### 2.3. SSS Signal Extractors

Equation (12) is the basis for estimating the internal and external components **b**_int_ and **b**_ext_ from given magnetic signal data **b**. That is, the least squares estimate $\hat{x}={\left[{\hat{\alpha }}^{T},{\hat{\beta }}^{T}\right]}^{T}$ is obtained as

(14) $$\begin{array}{c} \hat{x}={\left(S^{T}S\right)}^{-1}S^{T}b. \end{array}$$

Then, **b**_int_ and **b**_ext_ are estimated as

(15) $$\begin{array}{c} {\hat{b}}_{\mathrm{int}}=C\hat{\alpha }, \end{array}$$

(16) $$\begin{array}{c} {\hat{b}}_{\text{ext}}=D\hat{\beta }. \end{array}$$

We now derive SSS signal extractors and rewrite (15) and (16) using these extractors. To do so, let us define an operation to make a new column vector [*a*_*i*_,…,*a*_*j*_]^*T*^ by using the *i*th to *j*th components of **a** = [*a*_1_,…,*a*_*M*_]^*T*^ as [**a**]_[*i* : *j*]_ (namely, [**a**]_[*i* : *j*]_ = [*a*_*i*_,…,*a*_*j*_]^*T*^). From (14), we have

(17) $$\begin{array}{c} \hat{\alpha }={\left[{\left(S^{T}S\right)}^{-1}S^{T}b\right]}_{\left[1:N_{C}\right]}. \end{array}$$

With a small positive constant *κ*, the relationship

(18) $$\begin{array}{c} \hat{\alpha }={\left[{\left(S^{T}S\right)}^{-1}S^{T}b\right]}_{\left[1:N_{C}\right]}\approx {\left[{\left(S^{T}S+\kappa I\right)}^{-1}S^{T}b\right]}_{\left[1:N_{C}\right]}, \end{array}$$

holds. Then, using the matrix inversion formula

(19) $$\begin{array}{c} {\left(S^{T}S+\kappa I\right)}^{-1}S^{T}b=S^{T}{\left({SS}^{T}+\kappa I\right)}^{-1}b, \end{array}$$

we get

(20) $$\begin{array}{c} \hat{\alpha }\approx {\left[{\left(S^{T}S+\kappa I\right)}^{-1}S^{T}b\right]}_{\left[1:N_{C}\right]}={\left[S^{T}{\left({SS}^{T}+\kappa I\right)}^{-1}b\right]}_{\left[1:N_{C}\right]}=C^{T}{\left({SS}^{T}+\kappa I\right)}^{-1}b\approx C^{T}{\left({SS}^{T}\right)}^{-1}b=C^{T}{\left({CC}^{T}+{DD}^{T}\right)}^{-1}b. \end{array}$$

Using (15) and (20), we obtain

(21) $$\begin{array}{c} {\hat{b}}_{\mathrm{int}}\approx {CC}^{T}{\left({CC}^{T}+{DD}^{T}\right)}^{-1}b. \end{array}$$

Thus, the internal component **b**_int_ can be extracted by multiplying

(22) $$\begin{array}{c} P_{\mathrm{int}}={CC}^{T}{\left({CC}^{T}+{DD}^{T}\right)}^{-1}, \end{array}$$

with the magnetic-field data **b**. That is, the matrix **P**_int_ acts as a projector that passes the internal components and blocks the external ones (note that since (**P**_int_)^2^ = **P**_int_ and (**P**_int_)^*T*^ = **P**_int_ do not hold, **P**_int_ is not actually a projector). Therefore, we call **P**_int_ the SSS signal extractor in this paper.

In exactly the same manner, we can derive

(23) $$\begin{array}{c} {\hat{b}}_{\text{ext}}=D\hat{\beta }\approx {DD}^{T}{\left({CC}^{T}+{DD}^{T}\right)}^{-1}b, \end{array}$$

and the SSS external-signal extractor **P**_ext_ is derived as

(24) $$\begin{array}{c} P_{\text{ext}}={DD}^{T}{\left({CC}^{T}+{DD}^{T}\right)}^{-1}. \end{array}$$

This **P**_ext_ passes the external components but blocks the internal ones.

### 2.4. Interference Suppression

**Figure 1:**
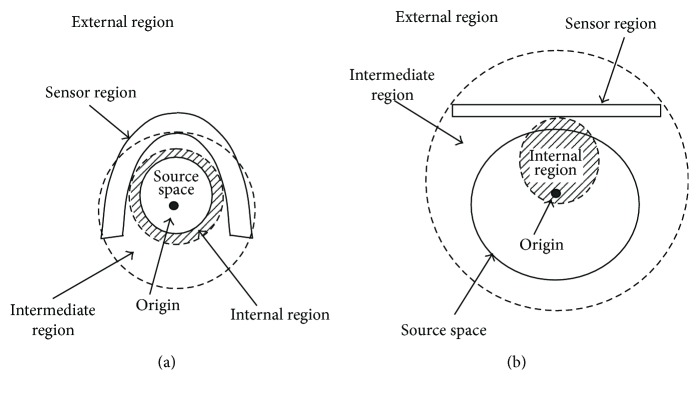
(a) A typical configuration of the internal region relative to the source space for a helmet-type sensor array. As can be seen, an appropriate location of the origin can be found such that the internal region covers the whole source space. (b) A possible configuration of the internal region relative to the source space in case of a flat sensor array. The internal region cannot entirely cover the source space, which extends into an intermediate region. In these figures, the inner circle with a broken line indicates the boundary between the internal and intermediate regions. The outer circle with a broken line indicates the boundary between the intermediate and external regions.

A key condition for the success of the SSS interference suppression is that the origin is properly set such that the source space *Ω* is included within the internal region and the interference sources are located within the external region. A typical configuration between the helmet-type sensor array and the source space is depicted in Figure 1(a). As can be seen in this figure, an appropriate location of the origin can be found so that the internal region covers the whole source space and the external region covers all locations of interference sources. (Note that, in this paper, interference indicates only environmental noise, and its sources are assumed to be located much farther from the sensors than the signal sources.)

When this key condition is met, (10) provides a natural separation between the signal and interference. That is, when the source space is included within the internal region, the relationship

(25) $$\begin{array}{c} b_{S}\in span\left(C\right), \end{array}$$

holds, where the notation span (**C**) indicates the span of the column vectors of **C**. This span (**C**) is referred to as the internal subspace in this paper. That is, since the signal vector **b**_*s*_ belongs to the internal subspace, **b**_*s*_ is expressed as a linear combination of the column vectors of **C**

(26) $$\begin{array}{c} b_{S}=\overset{N_{C}}{\underset{j=1}{\sum }}\alpha_{j}c_{j}=C\alpha , \end{array}$$

where **c**_*j*_ is the *j*th column of **C**, *α*_*j*_ is the *j*th expansion coefficient, and **α** is a column vector containing the coefficients (i.e., **α** = [*α*_1_,…,*α*_*N*_*C*__]^*T*^). Thus, denoting an *N*_*D*_ × 1 column vector whose elements are all zero by **0**, we can derive the relationship

(27) $$\begin{array}{c} P_{\mathrm{int}}b_{S}=P_{\mathrm{int}}C\alpha =P_{\mathrm{int}}S\left[\begin{array}{l} \alpha \\ 0 \end{array}\right]=C\left[C^{T}{\left({SS}^{T}\right)}^{-1}S\left[\begin{array}{l} \alpha \\ 0 \end{array}\right]\right]=C{\left[S^{T}{\left({SS}^{T}\right)}^{-1}S\left[\begin{array}{l} \alpha \\ 0 \end{array}\right]\right]}_{\left[1:N_{C}\right]}=C\left[{\left(S^{T}S\right)}^{-1}S^{T}S\left[\begin{array}{l} \alpha \\ 0 \end{array}\right]\right]=C\alpha =b_{S}. \end{array}$$

The equation above indicates that the SSS signal extractor **P**_int_ passes the signal vector **b**_*S*_ with no distortion.

The assumption that the interference sources are located within the external region leads to

(28) $$\begin{array}{c} b_{I}\in span\left(D\right), \end{array}$$

where span(**D**) is referred to as the external subspace. Since the interference vector **b**_*I*_ belongs to the external subspace, the interference vector **b**_*I*_ is expressed as

(29) $$\begin{array}{c} b_{I}=\overset{N_{D}}{\underset{j=1}{\sum }}\beta_{j}d_{j}=D\beta , \end{array}$$

where **d**_*j*_ is the *j*th column of **D**, *β*_*j*_ is the *j*th expansion coefficient, and **β** is a column vector containing coefficients **β** = [*β*_1_,…,*β*_*N*_*D*__]^*T*^ . Again denoting the *N*_*C*_ × 1 column vector whose elements are all zero by **0**, we have the relationship

(30) $$\begin{array}{c} P_{\mathrm{int}}b_{I}=P_{\mathrm{int}}D\beta =P_{\mathrm{int}}S\left[\begin{array}{l} 0\\ \beta \end{array}\right]=C{\left[{\left(S^{T}S\right)}^{-1}S^{T}S\left[\begin{array}{l} 0\\ \beta \end{array}\right]\right]}_{\left[1:N_{C}\right]}=C0=0. \end{array}$$

The equation above indicates that the extractor **P**_int_ completely blocks the interference vector **b**_*I*_.

Consequently, using (1) and (2), we show

(31) $$\begin{array}{c} P_{\mathrm{int}}y=P_{\mathrm{int}}b_{S}+P_{\mathrm{int}}+b_{I}+P_{\mathrm{int}}\varepsilon =b_{S}+\varepsilon^{\prime }. \end{array}$$

The equation above indicates that, by multiplying the extractor **P**_int_ with the data vector **y**, the signal vector **b**_*S*_ is selectively extracted with no distortion.

In (31), *ε*′ : (*ε*′ = **P**_int_*ε*) indicates the noise in the SSS-cleaned data. Assuming that the sensor noise *ε* is Gaussian with the covariance matrix *σ*^2^**I**, the covariance matrix of *ε*′, *Σ*_*ε*′_ is derived such that

(32) $$\begin{array}{c} \underset{\varepsilon^{\prime }}{\Sigma }=\langle \varepsilon^{\prime }{\left(\varepsilon^{\prime }\right)}^{T}\rangle =P_{\mathrm{int}}\langle {\varepsilon \varepsilon }^{T}\rangle P_{\mathrm{int}}^{T}=\sigma^{2}P_{\mathrm{int}}P_{\mathrm{int}}^{T}, \end{array}$$

where we have 〈*εε*^*T*^〉 = *σ*^2^**I** and the notation 〈·〉 indicates averaging. The equation above shows that **P**_int_**P**_int_^*T*^ can be considered as the noise gain of the SSS interference suppression process. Particularly, since the diagonal elements of **Σ**_*ε*′_ expresses the gain relationship between the variances of the input and output noises, we define the noise gain *G*_*ε*_ such that

(33) $$\begin{array}{c} G_{\varepsilon }=\frac{1}{M}\overset{M}{\underset{j=1}{\Sigma }}{\left[\underset{\varepsilon^{\prime }}{\Sigma }\right]}_{j,j}, \end{array}$$

where [Σ_*ε*′_]_*j*,*j*_ indicates the *j*th diagonal element of the matrix Σ_*ε*′_.

### 2.5. SSS Method for Flat Sensor Arrays

In Figure 1(b), a possible configuration of the internal region relative to the source space and sensors is depicted for the case of a flat sensor array. As shown here, the internal region may not cover the entire source space, and the source space is extended into the intermediate region. As a result, the lead field vector of a signal source, **l**_*q*_, may have components expanded by the columns of **D**, as well as components expanded by the columns of **C**, resulting in

(34) $$\begin{array}{c} l_{q}=C\alpha^{\prime }+D\beta^{\prime }, \end{array}$$

where **α**′ and **β**′ are column vectors containing the expansion coefficients.

Therefore, by applying the SSS extractor **P**_int_ to **l**_*q*_, we have

(35) $$\begin{array}{c} P_{\mathrm{int}}l_{q}=P_{\mathrm{int}}\left[C\alpha^{\prime }+D\beta^{\prime }\right]=C\alpha^{\prime }={\tilde{l}}_{q}. \end{array}$$

That is, the extractor **P**_int_ changes the lead field of this signal source from **l**_*q*_ to ${\tilde{l}}_{q}$. Assuming that *Q* signal sources exist, the signal vector **b**_*S*_(*t*) is expressed as

(36) $$\begin{array}{c} b_{S}\left(t\right)=\overset{Q}{\underset{q=1}{\sum }}s_{q}\left(t\right)l_{q}. \end{array}$$

This signal vector changes to ${\tilde{b}}_{S}\left(t\right)$, which is given by

(37) $$\begin{array}{c} {\tilde{b}}_{S}\left(t\right)=P_{\mathrm{int}}b_{S}\left(t\right)=P_{\mathrm{int}}\overset{Q}{\underset{q=1}{\sum }}s_{q}\left(t\right)l_{q}=\overset{Q}{\underset{q=1}{\sum }}s_{q}\left(t\right)P_{\mathrm{int}}l_{q}=\overset{Q}{\underset{q=1}{\sum }}s_{q}\left(t\right){\tilde{l}}_{q}. \end{array}$$

It is clear here that applying the SSS extractor **P**_int_ distorts the signal vector **b**_*S*_(*t*). This is a problem that occurs when the SSS method is applied to data measured with a flat sensor array. Computer simulation-based investigation of the signal distortion and its correction are given in Section 3.6.

As can be seen in Figures 1(a) and 1(b), the external region does not differ between helmet and flat sensor arrays. In this paper, we assume that all interference is environmental noise and no interference sources exist in the vicinity of sensors. Since, under this assumption, we assume that all interference sources are located in the external region, the interference vector **b**_*I*_(*t*) does not have components belonging to span (**C**), and applying **P**_int_ to the data vector removes the interference vector.

### 2.6. Evaluation of the SSS Method's Performance

As discussed in the preceding sections, a flat sensor array imposes nonideal conditions on the SSS interference suppression, and (25) is never fulfilled. In addition, the existence of sensor calibration errors (which will be considered in Section 3.4) could, to some extent, invalidate the assumption in (28). Therefore, for data from flat sensor arrays, the relationships

(38) $$\begin{array}{c} P_{\mathrm{int}}b_{S}=b_{S},\\ P_{\mathrm{int}}b_{I}=0, \end{array}$$

will never be attained.

We can evaluate the performance of the SSS method for data from flat sensor arrays by checking how close the SSS-processed results come to (38). Namely, we define the performance measures such that

(39) $$\begin{array}{c} G_{S}=\frac{\left\|P_{\mathrm{int}}b_{S}\right\|}{\left\|b_{S}\right\|}, \end{array}$$

(40) $$\begin{array}{c} G_{I}=\frac{\left\|P_{\mathrm{int}}b_{I}\right\|}{\left\|b_{I}\right\|}. \end{array}$$

In the equations above, *G*_*S*_ is called the signal gain. Ideally, *G*_*S*_ is equal to 1, and deviation of *G*_*S*_ from 1 is a measure of performance degradation of the SSS method. *G*_*I*_ is called the interference gain. Ideally, *G*_*I*_ is equal to zero, indicating that the method completely blocks the interference. Thus, a deviation of *G*_*I*_ from 0 is a measure of the performance degradation. Note that 1*/G_I_* has been often called the shield factor in the previous literature [9, 18]. We use *G*_*S*_ and *G*_*I*_ (or 1*/G_I_*, the shield factor), as well as the noise gain *G*_*ε*_ in (33) to evaluate the performance of the SSS method when it is applied to data from flat sensor arrays in Section 3.

## 3. Computer Simulation

### 3.1. Problems with Flat Sensor Data for SSS Applications

In order to show that the SSS method is still effective for data from sensors arranged on a flat plane, a series of computer simulation has been performed. First, we clarify the problems caused when the SSS method is applied to flat sensor data by comparing two cases of SSS application: one in which a helmet-type sensor array is used and the other in which a flat sensor array is used. For helmet-type sensor arrays, the key condition for the SSS method can be fulfilled, that is, one can find a proper location of the origin so that the internal region includes the source space and the external region includes the locations of interference sources, as depicted in Figure 1(a). Therefore, the SSS method can effectively suppress the interference with no signal distortion. For flat sensor arrays, however, the internal region covers only a part of the source space, which extends into the intermediate region, as depicted in Figure 1(b). Therefore, the signal vector has both external and internal components, and, as a result, the signal vector is distorted through the SSS application.

**Figure 2:**
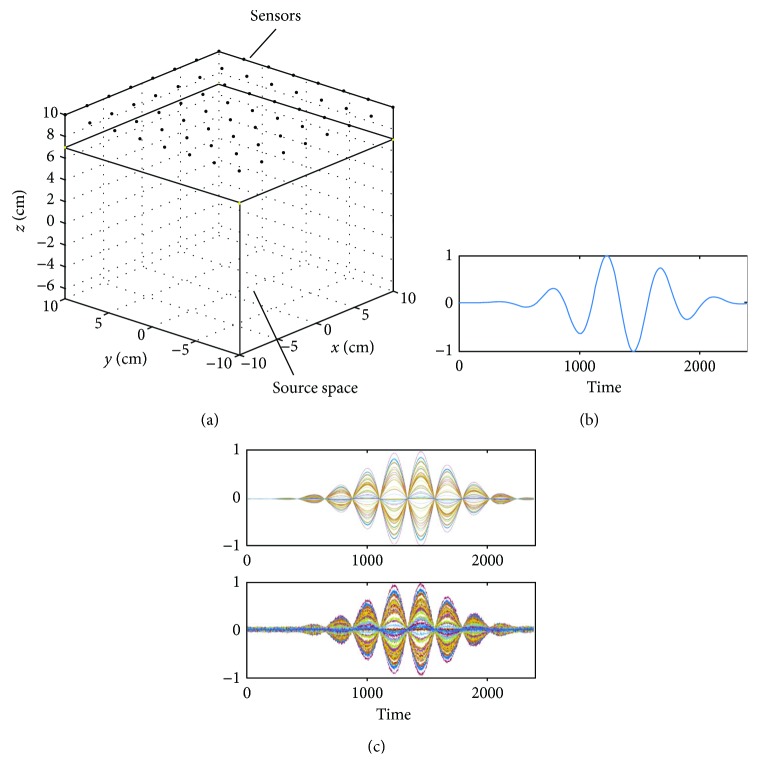
(a) An array of sensors and the coordinate system used in our computer simulation. The sensor array consists of 64 sensors arranged in an 8 × 8 configuration on the plane *z* = 10 cm, called the sensor plane. The sensor array covers an area of 20 cm × 20 cm, and the sensors measure the *z* component of the magnetic field, which is the component normal to the sensor plane. The source space (−10 ≤ *x* ≤ 10 cm, −10 ≤ *y* ≤ 10 cm, −7 ≤ *z* ≤ 7 cm) is shown. (b) The source time course assumed for a source located at (−3 cm, 0 cm, and 2 cm). (c) The signal sensor data **b**_*S*_(*t*) are shown in the upper panel, and the signal plus sensor-noise data **b**_*S*_(*t*) + *ε* are shown in the lower panel. Here, sensor noise was added to the signal data with the signal-to-noise ratio (SNR) of 10. The sensor time courses are normalized to the maximum value in each panel, and the ordinate indicates the normalized values.

Let us first check this fact. An array of sensors and the coordinate system used in our computer simulation are shown in Figure 2(a). The sensor array consists of 64 sensors arranged in an 8 × 8 configuration on the plane *z* = 10 cm; the plane on which sensors are arranged is called the sensor plane. The sensor array covers an area of 20 cm × 20 cm, and the sensors measure only the *z* component of the magnetic field, which is the component normal to the sensor plane. This sensor arrangement is almost the same as the one in the MC-6400MCG system (Hitachi High-Technologies Corporation, Tokyo, Japan), which is used in a number of investigations [11–13]. A similar sensor arrangement is used in the KRISS 64-channel biomagnetometer (Bio-Signal Research Center, KRISS, Daejeon, Korea). Therefore, the arrangement of the sensor array assumed in this computer simulation can be considered as a typical one used in current clinical MCG studies.

A single source was assumed to exist at (−3 cm, 0 cm, and 2 cm), and the source time course assigned to this source is shown in Figure 2(b). The signal sensor data **b***_S_*(*t*) were computed by projecting the source time course through the sensor lead field obtained using the Biot-Savart law. The signal data **b***_S_*(*t*) are shown in the upper panel of Figure 2(c). Sensor noise was then added to the signal data with a signal-to-noise ratio (SNR) of 10 to generate the signal plus sensor-noise data, **b**_*S*_(*t*) + *ε*, which are shown in the lower panel of Figure 2(c). Here, the sensor noise was assumed to be the white Gaussian noise uncorrelated between different sensor channels, that is, the noise vector had a statistical property of *ε* ~ *N*(*ε* | 0, *σ*^2^**I**), where *σ*^2^ is the variance of the noise in all sensor channels.

**Figure 3:**
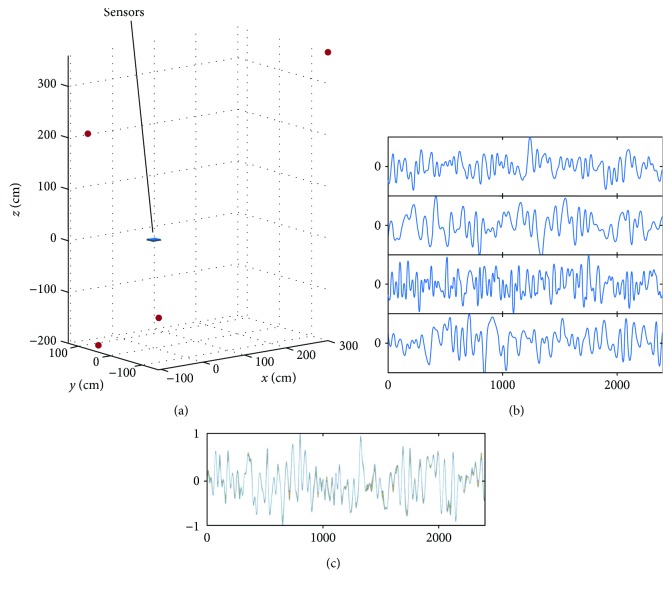
(a) Locations of four interference sources shown with respect to the sensors. (b) Random (normalized) time courses assigned to the four interference sources. (c) Generated interference sensor data **b**_*I*_(*t*). These interference sensor data are computed by projecting the interference-source time courses in (b) through the sensor lead field obtained using the Biot-Savart law. The sensor time courses are normalized, and the ordinate indicates the normalized values.

In order to generate environmental noise, four interference sources were assumed to exist; their coordinates and distances from the center of the sensor array are shown in Table 1. The locations of these interference sources with respect to the sensor array are shown in Figure 3(a). Four random time courses shown in Figure 3(b) were assigned to these four interference sources, and the interference data **b**_*I*_(*t*) were computed. The generated interference data are shown in Figure 3(c).

**Figure 4:**
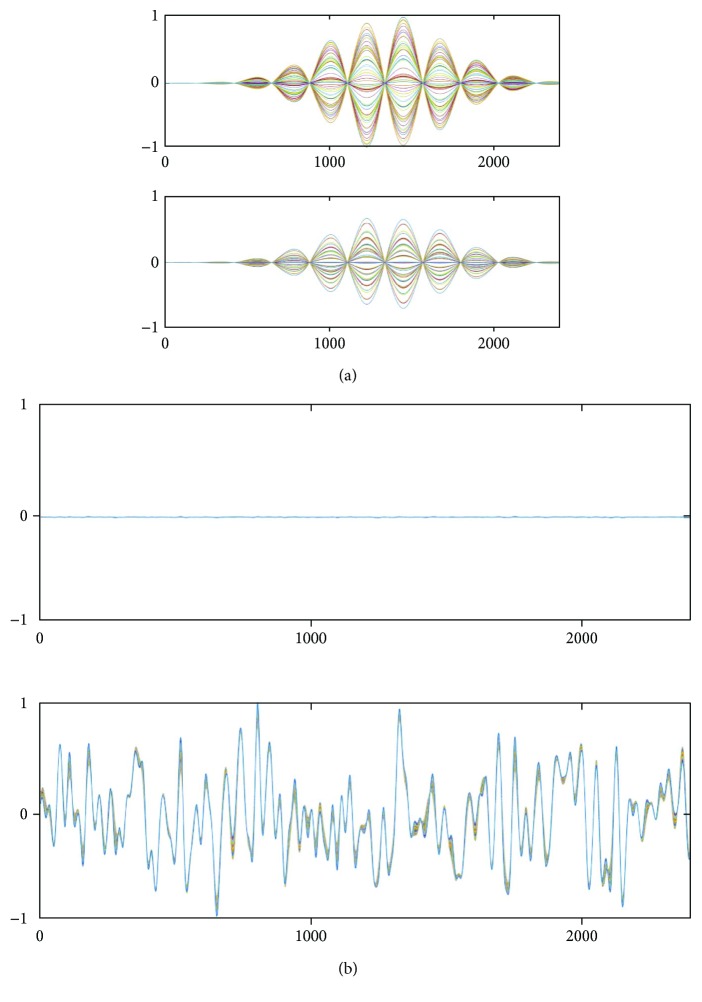
Results of experiments that apply the SSS extractors to **b**_*S*_(*t*) and **b**_*I*_(*t*) computed assuming the flat sensor array in Figure 2(a). (a) The upper and lower panels, respectively, show **P**_int_**b**_*S*_(*t*) and **P**_ext_**b**_*S*_(*t*), which indicate the internal and external components in **b**_*S*_(*t*). (b) The upper and lower panels, respectively, show **P**_int_**b**_*I*_(*t*) and **P**_ext_**b**_*I*_(*t*), which indicate the internal and external components in **b**_*I*_(*t*). In the sensor time courses in (a), they are normalized to the maximum value from the upper panel, and the sensor time courses in (b), they are normalized to the maximum value from the lower panel.

We applied the SSS extractors to **b**_*S*_(*t*) and **b**_*I*_(*t*) in order to check how large the internal and external components are in each of **b**_*S*_(*t*) and **b**_*I*_(*t*). The upper and lower panels, respectively, of Figure 4(a) show **P**_int_**b**_*S*_(*t*) and **P**_ext_**b**_*S*_(*t*). Here, **P**_int_**b**_*S*_(*t*) and **P**_ext_**b**_*S*_(*t*) indicate the internal and external components contained in **b**_*S*_(*t*). These results show that the signal vector **b**_*S*_(*t*) contains a considerable amount of external components, confirming the validity of our analysis in Section 2.5. The upper and lower panels, respectively, of Figure 4(b) show **P**_int_**b**_*I*_(*t*) and **P**_ext_**b**_*I*_(*t*). Here, **P**_int_**b**_*I*_(*t*) is nearly equal to zero, and **P**_ext_**b**_*I*_(*t*) is almost the same as **b**_*I*_(*t*). These results verify our arguments in Section 2.5 that **b**_*I*_(*t*) contains almost no internal components.

**Figure 5:**
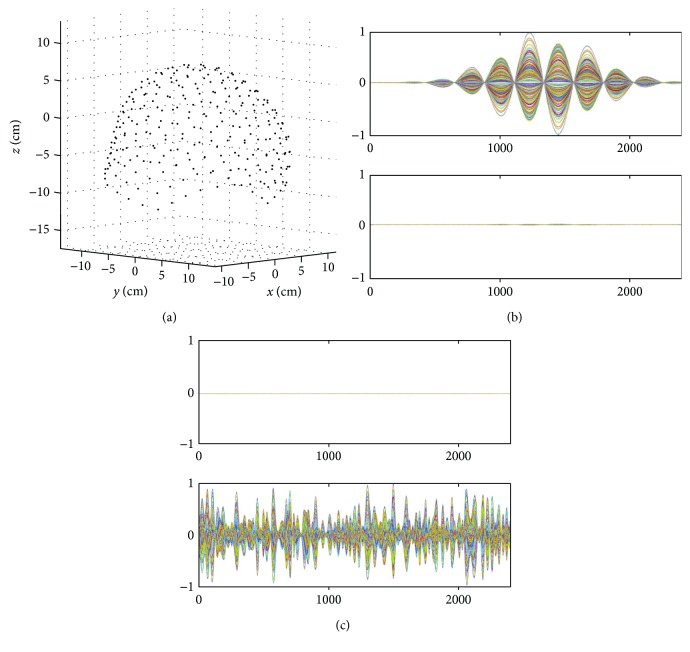
Results of the same experiments in which the SSS extractors are applied to **b**_*S*_(*t*) and **b**_*I*_(*t*), assuming a helmet-type sensor array used in MEG. (a) Locations of sensors in the helmet-type array assumed in this computer simulation. The sensor arrangement is from the 275-channel whole head sensor array of the Omega™ (VMS Medtech, Coquitlam, Canada). (b) The upper and lower panels, respectively, show **P**_int_**b**_*S*_(*t*) and **P**_ext_**b**_*S*_(*t*). (c) The upper and lower panels, respectively, show **P**_int_**b**_*I*_(*t*) and **P**_ext_**b**_*I*_(*t*). In the sensor time courses in (b), they are normalized to the maximum value from the upper panel, and the sensor time courses in (c), they are normalized to the maximum value from the lower panel.

We performed the same experiments using an MEG helmet-type sensor array for comparison; the arrangement of the helmet sensors used in this computer simulation is shown in Figure 5(a). The upper and lower panels, respectively, of Figure 5(b) show **P**_int_**b**_*S*_(*t*) and **P**_ext_**b**_*S*_(*t*), and the upper and lower panels, respectively, of Figure 5(c) show **P**_int_**b**_*I*_(*t*) and **P**_ext_**b**_*I*_(*t*). These results clearly confirm that the amount of the external components in **b**_*S*_(*t*), as well as the amount of the internal components in **b**_*I*_(*t*), is very small, explaining why the SSS method works well for data from helmet-type sensor arrays used in MEG.

### 3.2. Optimal Location of the Origin

**Figure 6:**
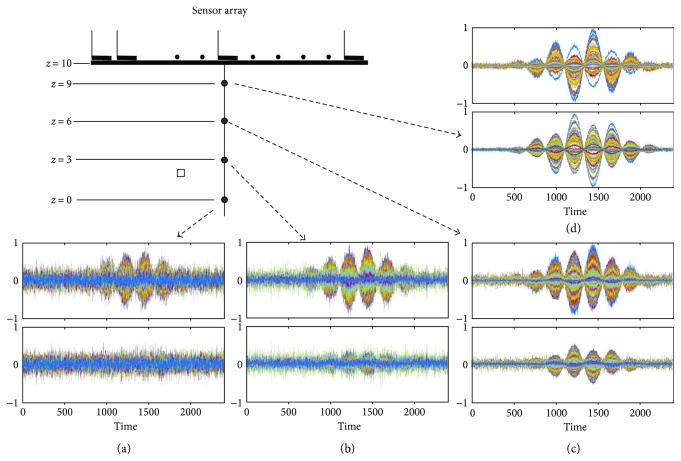
The internal component **P**_int_(**b**_*S*_(*t*) + *ε*) and the external component **P**_ext_(**b**_*S*_(*t*) + *ε*) of the signal vector (plus sensor noise) with four different locations of the origin (0, 0, and *z*_ori_): (a) *z*_ori_ = 0 cm, (b) *z*_ori_ = 3 cm, (c) *z*_ori_ = 6 cm, and (d) *z*_ori_ = 9 cm. The upper panel indicates the internal components, and the lower indicates the external components **P**_ext_**b**_*S*_(*t*). The filled circle shows the location of the origin, and the square indicates the location of the source. In each pair of the sensor time courses, they are normalized to the maximum value from the upper panel, and the ordinate indicates the normalized values.

We next explored the optimal location of the origin for SSS application to flat sensor data. The origin location significantly affects the final results of the SSS interference suppression, and, thus it is one of the most important parameters in the SSS implementation. In order to see how the origin location affects the SSS results, the internal component **P**_int_**b**_*S*_(*t*) and the external component **P**_ext_**b**_*S*_(*t*) were computed with the origin set at four different locations of (0, 0, and *z_ori_*) where *z*_ori_ was equal to 9 cm, 6 cm, 3 cm, and 0 cm. (Note that the center of the sensor array is located at (0 cm, 0 cm, and 10 cm)). Here, the signal sensor data (plus sensor noise) **b**_*S*_(*t*) + *ε* shown in Figure 2(c) were used. The truncation values *L*_*C*_ and *L*_*D*_ were, respectively, set at 7 and 3, which are the values found to be optimal in previous investigations [8, 9]. Results of this experiment are shown in Figure 6, exhibiting a general tendency that the signal leakage becomes larger when the origin becomes closer to the sensor plane (*z* = 10 cm). However, the results also show that when the origin becomes farther from the sensor plane, the SSS results become significantly noisy. In summary, the location of the origin affects both the amount of signal leakage and the noise in the SSS results.

**Figure 7:**
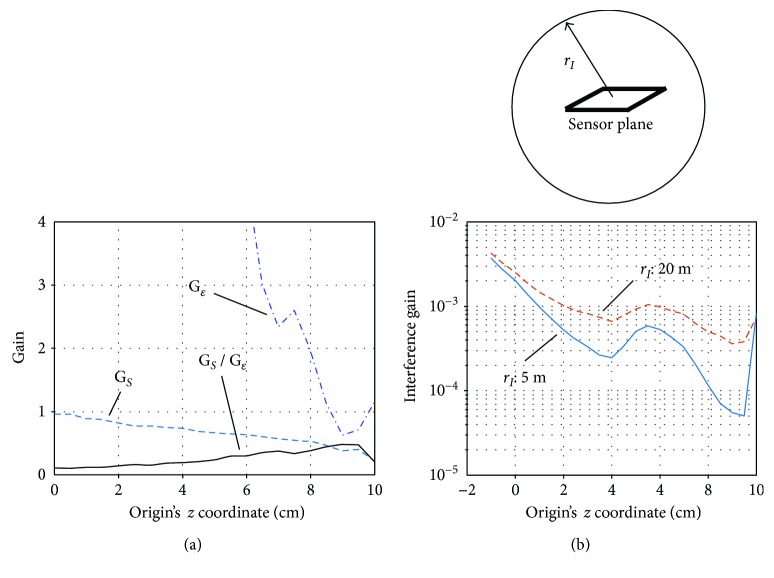
(a) Plots of the mean signal gain *G*_*S*_, the noise gain *G*_*ε*_, and their ratio *G*_*S*_/*G*_*ε*_ versus the *z* coordinate of the origin *z*_ori_. The signal sensor data **b**_*S*_(*t*) were computed by setting the signal source at one of the voxel locations, and the signal gain *G_S_* was obtained. The mean signal gain was computed by averaging *G_S_* obtained from all voxel locations. (b) The mean interference gain *G*_*I*_ versus *z*_ori_. The plot with the broken line shows the case *r*_*I*_ = 20 m, and the plot with the solid line shows the case *r*_*I*_ = 5 m. The interference data **b***_I_* were computed by setting a source at 100 equally spaced locations on the surface of a sphere with a radius of *r*_*I*_, and the interference gain *G*_*I*_ was obtained. The mean interference gain was computed by averaging *G*_*I*_ obtained from all 100 source locations. The sphere with a radius *r*_*I*_ is shown in the upper part.

The amount of signal leakage can be assessed by the signal gain *G*_*S*_ defined in (39). Let us derive a quantitative relationship of the signal gain *G*_*S*_ versus *z*_ori_. To do this, voxels with 0.5 cm intervals were assumed in a 3-dimensional source space (−10 ≤ *x* ≤ 10 cm, −10 ≤ *y* ≤ 10 cm, and −7 ≤ *z* ≤ 7 cm), which is shown in Figure 2(a). The signal sensor data **b**_*S*_(*t*) were computed by setting the signal source at one of the voxel locations, and the signal gain *G*_*S*_ was computed using **b**_*S*_(*t*). The mean signal gain was computed by averaging the signal gains obtained from all voxel locations. Here, the SSS extractor was derived with *z*_ori_ varied from 0 cm to 10 cm. The mean signal gain versus *z*_ori_ is plotted with a broken line in Figure 7(a). It can be seen in these plots that *G_S_* gradually decreases as the origin becomes closer to the sensor plane.

The noise gain *G*_*ε*_ is also plotted in Figure 7(a), and we can see that the noise gain becomes significantly larger as the origin becomes farther from the sensor plane, confirming the general tendency observed in Figure 6. The ratio *G*_*S*_/*G*_*ε*_ is also plotted in Figure 7(a), and it can be seen that the ratio gradually increases as the origin becomes closer to the sensor plane. It reaches maximum at *z*_ori_ approximately equal to 9 cm.

We next performed computer simulation to derive the relationship between the interference gain *G_I_* and the origin location *z*_ori_. To do this, we assumed a sphere with its radius equal to *r*_*I*_ and its center at the center of the sensor array; such a sphere is shown in the upper part of Figure 7(b). The surface of the sphere was defined as a region where interference sources exist, and the interference data **b**_*I*_(*t*) were computed by assuming that a single interference source exists on the surface. The interference gain *G*_*I*_ was computed with 100 different locations of the interference source, and the mean interference gain is computed and plotted with respect to the origin parameter *z*_ori_. Here, denoting the polar coordinate of the interference source by (*r*_*I*_, *θ*, *ϕ*), 100 locations of the interference source were determined as locations with 10 equal-interval *θ* and 10 equal-interval *φ*.

The plots of *G_I_* with respect to *z_ori_* are shown in Figure 7(b). Here, two cases, *r*_*I*_ = 5 m and *r*_*I*_ = 20 m, are shown. The case *r*_*I*_ = 5 m represents cases in which an interference source is located relatively near to the sensors, and the case *r*_*I*_ = 20 m represents cases in which an interference source is located relatively far from the sensors. These plots indicate that the interference gain generally decreases as the origin becomes closer to the sensors, and it reaches a minimum at *z*_ori_ ≈ 9 cm for both cases.

### 3.3. Choices of *L*_*C*_ and *L*_*D*_, Truncated Values of Multipole-Order ℓ

**Figure 8:**
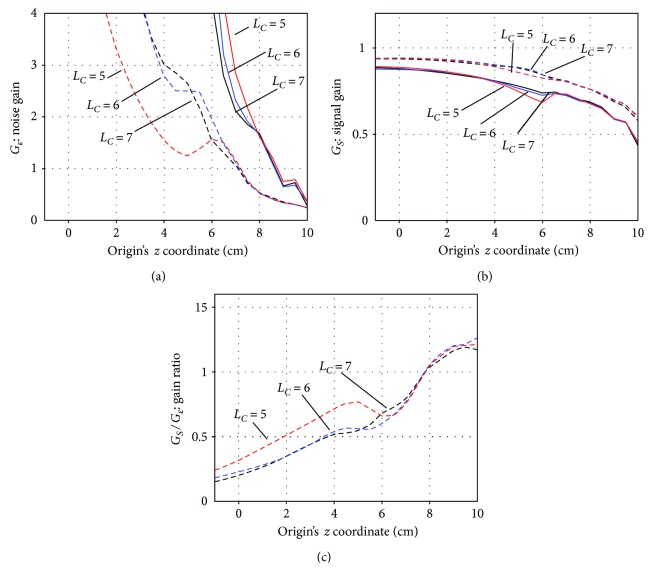
(a) The signal gain *G*_*S*_, (b) noise gain *G*_*ε*_, and (c) their ratio *G*_*S*_/*G*_*ε*_ plotted with respect to the origin's *z* coordinate, *z*_ori_, for three values of *L*_*C*_ (*L*_*C*_ = 5, 6, and 7) and two values of *L*_*D*_ (*L*_*D*_ = 2 and 3). The broken lines indicate the results with *L*_*D*_ = 2, and the solid lines indicate those with *L*_*D*_ = 3.

We explore the optimal values of the parameters *L*_*C*_ and *L*_*D*_, which are the truncation values of the multipole-order ℓ introduced in (10). So far, these parameters have been set at the values found to be optimal in previous investigations [8, 9], and so, *L*_*C*_ and *L*_*D*_ were, respectively, set at 7 and 3 in the preceding subsections. In Figure 8, the signal gain *G_S_*, noise gain *G_ε_*, and their ratio *G*_*S*_/*G*_*ε*_ are plotted with respect to *z*_ori_ for three different values of *L*_*C*_ (*L*_*C*_ = 5, 6, and 7) and two different values of *L*_*D*_ (*L*_*D*_ = 2 and 3). The noise gain is plotted in Figure 8(a), the signal gain in Figure 8(b), and the gain ratio in Figure 8(c). In these figures, the broken lines indicate the results with *L*_*D*_ = 2, and the solid lines indicate those with *L*_*D*_ = 3.

It can be seen that the noise gain is considerably smaller when *L*_*D*_ = 2 than when *L*_*D*_ = 3. The signal gain when *L*_*D*_ = 2 is greater than when *L*_*D*_ = 3. The differences in the signal and noise gains among different values of *L_C_* are generally small. These results suggest that *L*_*D*_ should be set at 2, rather than 3. In Figure 8(c), the plots of the gain ratio *G*_*S*_/*G*_*ε*_ for *L*_*D*_ = 2 are shown to have peak maxima when *z*_ori_ *>* 8, regardless of the value of *L*_*C*_. The results in Figure 8 suggest that, as far as the signal and noise gains concerned, *L*_*D*_ = 2 should be the best choice, and any value of *L*_*C*_ = 5, 6, and 7 may be chosen if we set *z*_ori_ such that *z*_ori_ *>* 8.

**Figure 9:**
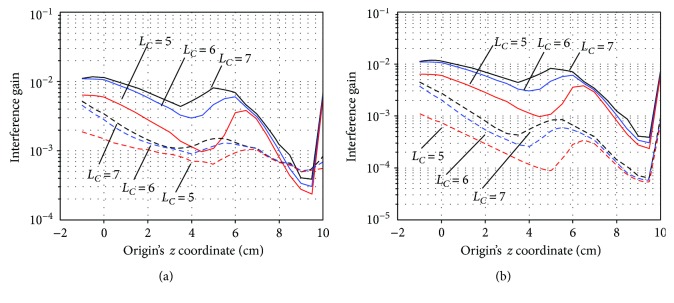
The interference gain plotted with respect to the origin's *z* coordinate, *z*_ori_, for three values of *L*_*C*_ (*L*_*C*_ = 5, 6, and 7) and two values of *L*_*D*_ (*L*_*D*_ = 2 and 3). (a) The distance to the interference source *r*_*I*_ is set at 5 m. (b) The distance to the interference source *r*_*I*_ is set at 20 m. The broken lines indicate the results with *L*_*D*_ = 2, and the solid lines indicate those with *L*_*D*_ = 3.

The interference gain is plotted with respect to *z*_ori_ for the three values of *L*_*C*_ and the two values of *L*_*D*_ in Figure 9. Here, the cases *r*_*I*_ = 5 m and *r*_*I*_ = 20 m are, respectively, shown in Figures 9(a) and 9(b), where the broken lines indicate the results of *L*_*D*_ = 2 and the solid lines indicate those of *L*_*D*_ = 3. These plots show that regardless of the values of *L*_*C*_ and *L*_*D*_, there is a general tendency that the gain decreases as the origin becomes closer to the sensors, and it reaches a minimum near *z*_ori_ equal to 9 cm. Namely, the value of *z*_ori_ ≈ 9 cm gives the minimum interference gain (the maximum shield factor) for all values of *L*_*C*_ and *L*_*D*_. On the basis of the results in Figures 8 and 9, we can conclude that the origin parameter *z*_ori_ should be set at 9, that is, the best origin location is determined as (0, 0, and 9), which is 1 cm below the center of the sensor array. We use this value throughout the experiments described below.

### 3.4. Influence of Sensor Calibration Errors

The SSS method is known to be very sensitive to sensor calibration errors, and an accurately calibrated sensor array is needed for effective suppression of interference [18]. Sensor calibration errors are known to severely affect the shielding capability, so the influence of sensor calibration errors on the interference gain *G*_*I*_ is investigated by using erroneous sensor locations and orientations when computing the SSS basis vectors. Here, the sensor location error *E*_loc_ is assessed using

(41) $$\begin{array}{c} E_{loc}=\left(\frac{1}{M}\right)\overset{M}{\underset{j=1}{\sum }}\frac{\left\|r_{j}^{d}-{\hat{r}}_{j}^{d}\right\|}{\left\|{\hat{r}}_{j}^{d}\right\|}, \end{array}$$

where **r**_*j*_^*d*^ is the true location of the *j*th sensor, and ${\hat{r}}_{j}^{d}$ is the calibrated location of this sensor. Note that ${\hat{r}}_{j}^{d}$ is assumed to contain an error. Here, **r**_*j*_^*d*^ is obtained by adding (small) random displacements to ${\hat{r}}_{j}^{d}$. The signal vector **b**_*S*_(*t*) and the interference vector **b**_*I*_(*t*) were computed using the true location **r**_*j*_^*d*^, while the SSS basis vectors were computed using the calibrated location ${\hat{r}}_{j}^{d}$. In exactly the same manner, the error in the sensor orientation, *E*_ori_, is assessed using

(42) $$\begin{array}{c} E_{ori}=\left(\frac{1}{M}\right)\overset{M}{\underset{j=1}{\sum }}\frac{\left\|\zeta_{j}-{\hat{\zeta }}_{j}\right\|}{\left\|{\hat{\zeta }}_{j}\right\|}, \end{array}$$

where **ζ***_j_* is the true normal vector of the *j*th sensor, and **ζ**_**b***j*_ is the calibrated normal vector, which is ${\hat{\zeta }}_{j}=\left(0,0,1\right)$. Here, **ζ**_*j*_ is obtained by adding random vectors to ${\hat{\zeta }}_{j}$. The signal vector **b**_*S*_(*t*) and the interference vector **b**_*I*_(*t*) were computed using **ζ**_*j*_, while the SSS basis vectors were computed using the erroneous orientation ${\hat{\zeta }}_{j}$.

**Figure 10:**
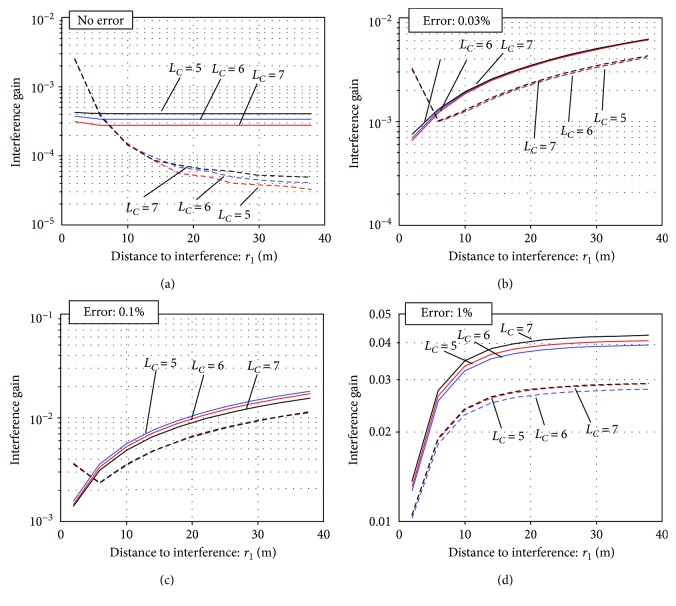
The plots of the interference gain *G_I_* with respect to the distance to the interference source, *r*_*I*_, for the three values of *L*_*C*_ and the two values of *L*_*D*_. (a) No calibration errors (*E*_Loc_ = *E*_Ori_ = 0). (b) Calibration errors of *E*_Loc_ = *E*_Ori_ = 0.03%. (c) Calibration errors of *E*_Loc_ = *E*_Ori_ = 0.1%. (d) Calibration errors of *E*_Loc_ = *E*_Ori_ = 1%. The broken lines indicate the results with *L*_*D*_ = 2 and the solid lines indicate those with *L*_*D*_ = 3. The origin was set at (0 cm, 0 cm, and 9 cm) (*z*_ori_ = 9 cm).

In Figure 10, the interference gain is plotted with respect to *r*_*I*_, the distance to the interference source, for the three values of *L*_*C*_ and the two values of *L*_*D*_. Again, the broken lines indicate the results with *L*_*D*_ = 2, and the solid lines indicate those with *L*_*D*_ = 3. Here, the plots in Figure 10(a) indicate the case of no calibration error (*E*_loc_ = *E*_ori_ = 0). The plots in Figures 10(b)–10(d), respectively, indicate the cases *E*_loc_ = *E*_ori_ = 0.03%, *E*_loc_ = *E*_ori_ = 0.1%, and *E*_loc_ = *E*_ori_ = 1%. In Figures 10(b)–10(d), the mean values obtained over 100 Monte Carlo trials are plotted. That is, a set of random values were assigned as the errors of sensor locations and orientations in each trial, and the mean results from 100 such trials are plotted.

First, we can observe that the interference gain (i.e., the shield factor) is seriously affected by the calibration errors. When there are no calibration errors, the shield factor 1*/G_I_* for *r_I_ >* 15 m is greater than 10^4^ with *L*_*D*_ = 2 but it drops to less than 30 when *E*_loc_ = *E*_ori_ = 1%. Regarding the optimal choices of the parameters *L*_*C*_ and *L*_*D*_, the choice of *L*_*D*_ = 2 mostly gives greater shield factor than *L*_*D*_ = 3. There are no significant differences among the choices of *L*_*C*_, but the choice of *L*_*C*_ = 6 gives slightly better results when *E*_loc_ = *E*_ori_ > 0.1%. Since such errors as *E*_loc_ = *E*_ori_ > 0.1% may be considered the practical values of sensor calibration errors, the choices of *L*_*C*_ = 6 and *L*_*D*_ = 2 are determined as the best choices for the truncation of the multipole parameter ℓ. Note that, according to the arguments for signal and noise gains in Section 3.3, the choices of *L*_*C*_ = 6 and *L*_*D*_ = 2 also give the best results.

**Figure 11:**
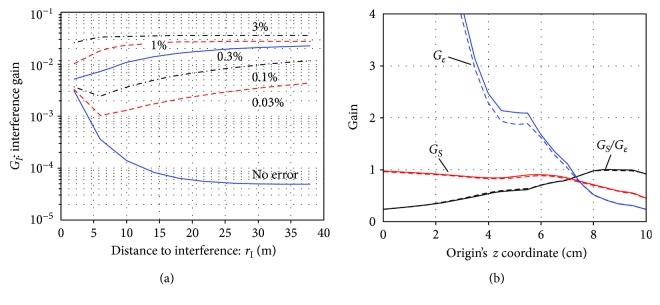
(a) The interference gain *G*_*I*_ replotted with respect to *r*_*I*_, the distance to interference sources. The choices of *L*_*C*_ = 6, *L*_*D*_ = 2, and *z*_ori_ = 9 cm were used. (b) The signal and noise gains versus the origin's *z* coordinate (*z*_ori_). The plots with the solid line indicate the case of no calibration errors, and the plots with the broken line indicate the case of 1% calibration errors. The multiple-order truncation was set such that *L*_*C*_ = 6 and *L*_*D*_ = 2.

With the choices of *L*_*C*_ = 6, *L*_*D*_ = 2, and *z*_ori_=9 cm, the interference gain *G*_*I*_ is replotted with respect to *r*_*I*_, the distance to the interference source. The results are shown in Figure 11(a). It can be seen that the shield factor (1*/G_I_*) exceeds 10^4^ for *r*_*I*_ > 15 m when no calibration errors exist but that the shield factor drops to 100 when calibration errors of 0.1% exist, and to 30 when calibration errors of 1% exist. With the parameter settings, *L*_*C*_ = 6, *L*_*D*_ = 2, and 1% sensor calibration errors, signal and noise gains versus *z*_ori_ are plotted in Figure 11(b). Here, the plot with the solid line indicates the case of no calibration errors, and the plot with the broken line indicates the case of 1% calibration errors. The two plots nearly overlap, and this fact confirms that the influence of calibration errors on the signal and noise gains is small.

### 3.5. The Performance of the SSS Method for Different Sensor Arrays

Here, the SSS performance with two different types of flat sensor arrays is tested: one is a sensor array with a larger sensor coverage and the other is a sensor array consisting of vector sensors. In the sensor array with a larger coverage, the sensors are arranged on a 24 cm × 24 cm coverage area and consist of 10 × 10 sensors that measure the magnetic field normal to the sensor plane. This sensor array is a larger-scale version of the sensor array used in the preceding subsections. The vector sensor array consists of 6 × 6 vector sensors arranged on a 20 cm × 20 cm coverage area. Here, a vector sensor indicates a set of three sensors; each measures one of the *x*, *y*, and *z* components of the magnetic field, and therefore this sensor array actually has a total of 108 sensors.

**Figure 12:**
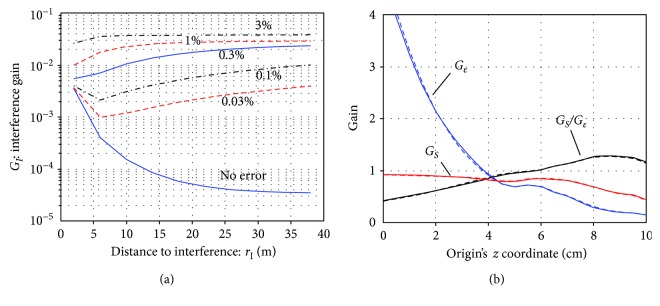
The SSS performance computed by assuming a larger sensor array in which the sensors are arranged on a 24 cm × 24 cm coverage area and consist of 10 × 10 sensors that measures the magnetic field normal to the sensor plane. (a) The interference gain *G*_*I*_ plotted with respect to *r*_*I*_ for six different calibration errors. The choices of *L*_*C*_ = 6, *L*_*D*_ = 2, and *z*_ori_ = 9 cm were used. (b) The signal and noise gains versus the origin's *z* coordinate (*z*_ori_). The plots with the solid line indicate the case of no calibration errors, and the plots with the broken line indicate the case of 1% calibration errors. The multiple-order truncation was set such that *L*_*C*_ = 6 and *L*_*D*_ = 2.

Assuming the sensor array with a larger sensor coverage, the interference gain *G*_*I*_ versus *r*_*I*_ (the distance to the interference sources) was plotted in Figure 12(a). It can be seen that the plots in this figure are almost the same as the plots in Figure 11(a), suggesting that the influence of the number of sensors and sensor coverage on the shielding capability is rather small. The signal and noise gains with respect to the origin's *z* coordinate (*z*_ori_) were plotted in Figure 12(b), where the plot with the solid line indicates the case of no calibration errors and the plot with the broken line indicates the case of 1% calibration errors. Again, we can observe that the plots here are very similar to the plots in Figure 11(b).

**Figure 13:**
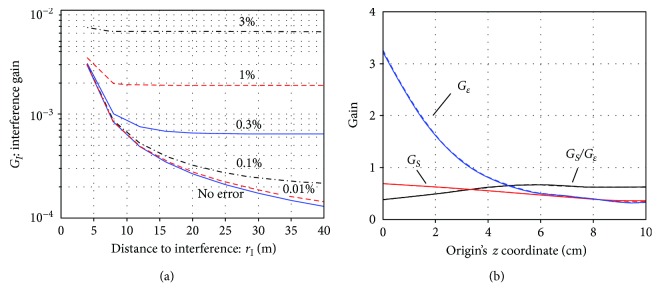
The SSS performance computed by assuming a vector sensor array in which the array consists of 6 × 6 vector sensors arranged on a 20 cm × 20 cm coverage area. (a) The interference gain *G_I_* plotted with respect to *r_I_* for six different calibration errors. The choices of *L*_*C*_ = 6, *L*_*D*_ = 2, and *z*_ori_ = 9 cm were used. (b) The signal and noise gains versus the origin's *z* coordinate (*z*_ori_). The plots with the solid line indicates the case of no calibration errors, and the plots with the broken line indicates the case of 1% calibration errors. The multiple-order truncation was set such that *L*_*C*_ = 6 and *L*_*D*_ = 2.

The SSS performance for the vector sensor array was investigated. The interference gain *G_I_* versus *r_I_* was plotted in Figure 13(a). We can observe significant improvements in the shielding capability.

That is, with 1% calibration errors, the vector sensor array has a shield factor of approximately 500 (*G*_*I*_ = 2 × 10^−3^). The two sensor arrays with normal-component-only sensors have shield factors of nearly 30 with 1% calibration errors, according to Figures 11(a) and 12(a). These results are consistent with the previous investigation [19], which have reported SSS performance improvements due to the use of tangential sensors. The signal and noise gains with respect to the origin's *z* coordinate (*z*_ori_) are plotted for the vector sensor array in Figure 13(b). It can be seen that the plots here are very similar to the plots in Figure 11(b)or 12(b), indicating that these gains are not affected by the use of vector sensors.

### 3.6. Correction of Signal Distortion in Source Space Analysis

#### 3.6.1. Two-Dimensional Current-Density Reconstruction Experiments

**Figure 14:**
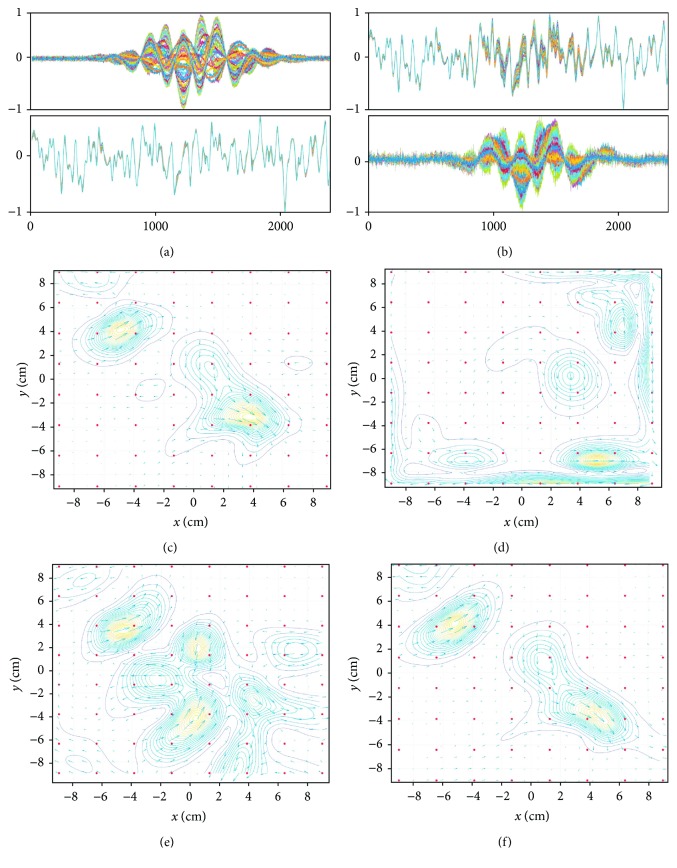
(a) Upper panel: the signal plus sensor-noise time courses **b**_*S*_(*t*) + *ε*. Lower panel: the interference time courses **b**_*I*_(*t*). (b) Upper panel: the interference-overlapped sensor time courses **y**(*t*). Lower panel: the SSS interference removal results **P**_int_**y**(*t*). These time courses are normalized to the maximum value in each panel. (c) The current-density map obtained from **b**_*S*_(*t*) + *ε*. (d) The current-density map obtained from interference-overlapped sensor time courses **y**(*t*). (e) The current-density map obtained from the SSS interference-removal results **P**_int_**y**(*t*) with the original lead field **L**(**r**). (f) The current-density map obtained using **P**_int_**y**(*t*) with the SSS-modified lead field $\tilde{L}\left(r\right)=P_{\mathrm{int}}L\left(r\right)$. Here, two-dimensional current-density maps on the plane *z* = 2 cm, which is the plane 8 cm below the sensor plane, are reconstructed using the field map at *t* = 1200. The signal data **b**_*S*_(*t*) were computed assuming two sources, located at (−5 cm, 4 cm, and 3 cm) and (4 cm, −3 cm, and 3 cm).

As discussed in Section 2.5, the SSS-processed signal vector ${\tilde{b}}_{S}\left(t\right)$$\left({\tilde{b}}_{S}\left(t\right)=P_{\mathrm{int}}b_{S}\left(t\right)\right)$ becomes distorted because the SSS application causes the removal of the external components from the signal vector **b**_*S*_(*t*). This distortion, however, can be corrected by using the SSS-modified lead field because the distorted signal vector is expressed as a sum of distorted lead field vectors, as shown in (37). A series of computer simulations were performed to verify this idea. The results for 2-dimensional current-density mapping are presented in Figure 14.

The signal data **b**_*S*_(*t*) were computed assuming the two sources located at (−5 cm, 4 cm, 3 cm) and (4 cm, −3 cm, and 3 cm). The signal plus sensor-noise data **b**_*S*_(*t*) + *ε* are shown in the upper panel of Figure 14(a). The interference **b***_I_*(*t*) is generated by assuming the same four sources as in Figure 3(a); the generated interference is shown in the lower panel of Figure 14(a). The upper panel of Figure 14(b) shows the sensor time courses **y**(*t*); **y**(*t*) = **b**_*S*_(*t*) + *ε* + *ξ ***b**_*I*_(*t*), where a positive constant *ξ* controls the signal-to-interference ratio (SIR) (The SIR is defined as the ratio 〈‖**b**_*S*_(*t*)‖〉/〈‖*ξ ***b**_*I*_(*t*)‖〉 in this computer simulation), which was set equal to 0.25 in this computer simulation. The SSS interference suppression results **P**_int_**y**(*t*) are shown in the lower panel of Figure 14(b).

The current-density reconstruction was performed using RENS beamformer, proposed in [20, 21]. The current-density map obtained from the signal plus sensor-noise data, **b**_*S*_(*t*) + *ε*, is shown in Figure 14(c). This map works as the ground truth for the following experiments. The current-density map obtained from the interference-overlapped data **y**(*t*) is shown in Figure 14(d). The results far from the ground truth are obtained due to the overlap of the large interference.

The current-density reconstruction was performed twice, using the SSS results **P**_int_**y**(*t*) with the original lead field **L**(**r**) and with the SSS-modified lead field $\tilde{L}\left(r\right)$: $\tilde{L}\left(r\right)=P_{\mathrm{int}}L\left(r\right)$. The current-density map obtained with the original lead field **L**(**r**) is shown in Figure 14(e). A considerable amount of distortion can be seen in this current-density map, although the SSS method seems to have nearly perfectly removed the interference according to the SSS-processed time courses (the lower panel of Figure 14(b)). The current-density map obtained with the SSS-modified lead field is shown in Figure 14(f). Here, results very close to the ground truth can be obtained; these results verify the idea that the signal distortion can be compensated for by using the SSS-modified lead field in the voxel space analysis.

#### 3.6.2. Three-Dimensional Source Localization Experiments

**Figure 15:**
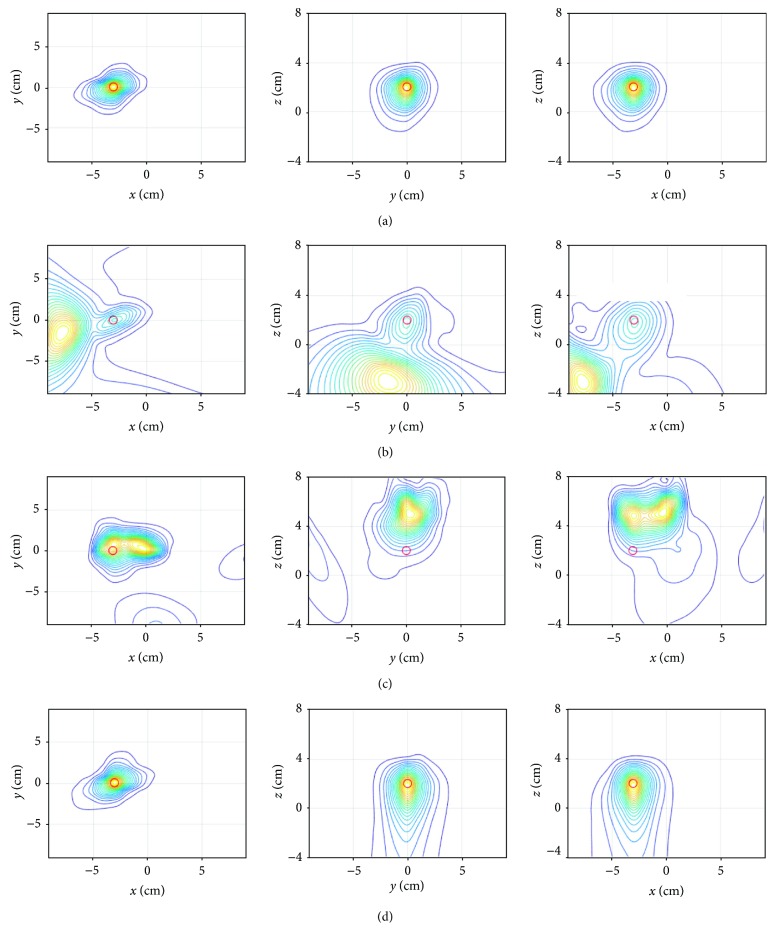
(a) Source reconstruction results obtained using the signal plus sensor-noise time courses in Figure 2(c). (b) Source reconstruction results obtained using the sensor time courses **y**(*t*) created by adding the interference data shown in Figure 3(c) onto the signal plus sensor-noise time courses in Figure 2(c). (c) Source reconstruction results obtained using the SSS interference removal results **P**_int_**y**(*t*) with the original lead field **L**(**r**). (d) Source reconstruction results obtained using the SSS interference removal results **P**_int_**y**(*t*) with the SSS-modified lead field $\tilde{L}\left(r\right)$. The RENS beamformer [20, 21] was applied to the field data at *t* = 1260 for three-dimensional source reconstruction.

Next, three-dimensional source localization experiments were performed. Reconstruction results obtained using the signal plus sensor-noise data shown in Figure 2(c) are shown in Figure 15(a). A single source is reconstructed near (−3, 0, and 2), which is the location assumed for the data generation. These results work as the ground truth when evaluating the following results. The interference-overlapped sensor data **y**(*t*) were computed using the interference data shown in Figure 3(c) overlapped onto these signal plus sensor-noise data with the SIR equal to 0.25. The source reconstruction was performed using the interference-overlapped sensor data **y**(*t*), and the results are shown in Figure 15(b). A large influence from the interference can be seen here.

The source reconstruction was carried out using the SSS interference suppression results **P**_int_**y**(*t*) with the original lead field **L**(**r**). The results are shown in Figure 15(c), in which a single source is reconstructed but the location of the source differs considerably from the assumed location. The source reconstruction was then carried out using the SSS-modified lead field $\tilde{L}\left(r\right)$, and the results are shown in Figure 15(d). These results are almost the same as the ground truth, verifying the effectiveness of the use of the SSS-modified lead field in the voxel space analysis.

## 4. Discussion and Summary

This paper presented computer simulation-based investigation to explore the possibility of applying the SSS method to data measured from an array of sensors arranged on a flat plane, which is commonly used in magnetocardiographic applications. The findings from the investigation are summarized as follows:

1. When applying the SSS method to data from a flat sensor array, a signal vector has components of the external subspace as well as those of the internal subspace. As a result, the signal is distorted through the SSS interference suppression process.
2. The signal distortion can be compensated for by using the SSS-modified lead field in voxel space analysis. The computer simulations using two-dimensional current-density mapping and three-dimensional source localization confirmed that the distortion can be corrected in the voxel space.
3. The origin location can significantly affect the results of the SSS method. It is shown that the optimal location of the origin can be determined by assessing the dependence of signal and noise gains of the SSS extractor on the origin location. The optimal location is empirically found to be approximately 1 cm below the sensor plane for typical flat sensor arrays used in MCG applications.
4. The optimal values of the parameters *L*_*C*_ and *L*_*D*_, the truncation values of the multipole-order ℓ, can also be determined by evaluating dependences of the signal, noise, and interference gains (shield factor) on these parameters. Results of computer simulation suggest *L*_*D*_ = 6 and *L*_*D*_ = 2 to be the optimal choices for typical flat sensor arrays currently used in clinical magnetocardiography.
5. The sensor calibration errors affect the shielding capability. The shield factor exceeds 10^4^ for interference originating from fairly distant sources (*r*_*I*_ > 15 m) when no calibration errors exist. However, the shield factor drops to approximately 100 when the calibration errors become 0.1% and to 30 when the calibration errors become 1%.
6. The shielding capability can significantly be improved by using vector sensors, which measure the *x*, *y*, and *z* components of the magnetic field. With 1% calibration errors, a vector sensor array still maintains a shield factor of approximately 500, while the arrays with sensors measuring only the normal direction have a shield factor of about 30 with the same 1% calibration errors.

Finally, it should be emphasized that the SSS interference suppression method is effective even for arrays of sensors arranged on a flat plane. This is the main finding in this paper. The use of such signal processing methods with low-cost sensors, such as magnetoresistive sensors, can lead to the development of low-initial-cost and maintenance-free magnetocardiography systems, which in the near future may replace the electrocardiogram now routinely used in hospitals.

## Figures and Tables

**Table 1:** Locations of interference sources assumed in a computer simulation.

Source number	Location (cm)	Distance from the sensor array (cm)
1	(−60, 130, 200)	238
2	(300, −150, 360)	485
3	(−72, −80, −200)	236
4	(−105, −150, −100)	214
